# Blockchain Technology Projects to Provide Telemedical Services: Systematic Review

**DOI:** 10.2196/17475

**Published:** 2021-08-18

**Authors:** Konstantin Koshechkin, Georgy Lebedev, George Radzievsky, Ralf Seepold, Natividad Madrid Martinez

**Affiliations:** 1 Federal State Autonomous Educational Institution of Higher Education I.M. Sechenov First Moscow State Medical University of the Ministry of Health of the Russian Federation (Sechenov University) Moscow Russian Federation; 2 Federal Research Institute for Health Organization and Informatics Moscow Russian Federation; 3 Ubiquitous Computing Lab HTWG Konstanz Konstanz Germany; 4 IoTLab Reutlingen University Reutlingen Germany

**Keywords:** telemedicine, distributed ledger, health information exchange, blockchain

## Abstract

**Background:**

One of the most promising health care development areas is introducing telemedicine services and creating solutions based on blockchain technology. The study of systems combining both these domains indicates the ongoing expansion of digital technologies in this market segment.

**Objective:**

This paper aims to review the feasibility of blockchain technology for telemedicine.

**Methods:**

The authors identified relevant studies via systematic searches of databases including PubMed, Scopus, Web of Science, IEEE Xplore, and Google Scholar. The suitability of each for inclusion in this review was assessed independently. Owing to the lack of publications, available blockchain-based tokens were discovered via conventional web search engines (Google, Yahoo, and Yandex).

**Results:**

Of the 40 discovered projects, only 18 met the selection criteria. The 5 most prevalent features of the available solutions (N=18) were medical data access (14/18, 78%), medical service processing (14/18, 78%), diagnostic support (10/18, 56%), payment transactions (10/18, 56%), and fundraising for telemedical instrument development (5/18, 28%).

**Conclusions:**

These different features (eg, medical data access, medical service processing, epidemiology reporting, diagnostic support, and treatment support) allow us to discuss the possibilities for integration of blockchain technology into telemedicine and health care on different levels. In this area, a wide range of tasks can be identified that could be accomplished based on digital technologies using blockchains.

## Introduction

In terms of work organization, the health care system has not changed much over the past century. Simultaneously, the technological infrastructure used by people has undergone complete digitalization, with significant changes occurring over the past decades. Blockchain technology has already existed for more than 10 years; however, it did not affect health care in general. Simultaneously, it was clearly shown that telemedicine could significantly affect clinical outcomes in several areas [1].

Interest in using blockchain technology for health care systems has become prominent since 2017. For example, a study conducted in the United States showed high levels of interest in these solutions among medical service consumers. Approximately 19% of the responding hospital executives and 80% of the payers were either considering or were in the process of implementing blockchain solutions [2].

The purpose of this work is to summarize and systematize the methodology for applying blockchain technology in providing telemedicine services. For implementing blockchain technology in telemedicine, surveys were conducted to obtain answers the following questions. How can blockchain-based projects improve telemedicine services? What are the most common features of these solutions?

Telemedical services at the present level of operation allow remote consultations with doctors and consultations of medical workers with patients using communication media such as the Internet. Doctors can monitor patients' health statuses remotely using medical sensors based on the principles of the Internet of Things (IoT). Moreover, doctors can write electronic prescriptions [3,4].

In the future, blockchain technology could potentially help obtain personalized, authentic, and secure health care by merging the entire real-time clinical data of a patient and presenting it in an up-to-date secure health care setup [5,6].

The medical services market is very conservative, and introducing new technologies requires a long time. However, the significant advantages of the data transfer medium and long-term projects can lead to a radical transformation of the health care system as a whole. The provision of telemedicine services can be the main focus of blockchain technology implementation. Solutions for the decentralized distribution of health information constitute one of the most significant benefits of telemedicine [7-9].

Public blockchain is simultaneously a peer-to-peer network and a public database without a central server [10].

**Figure 1:**
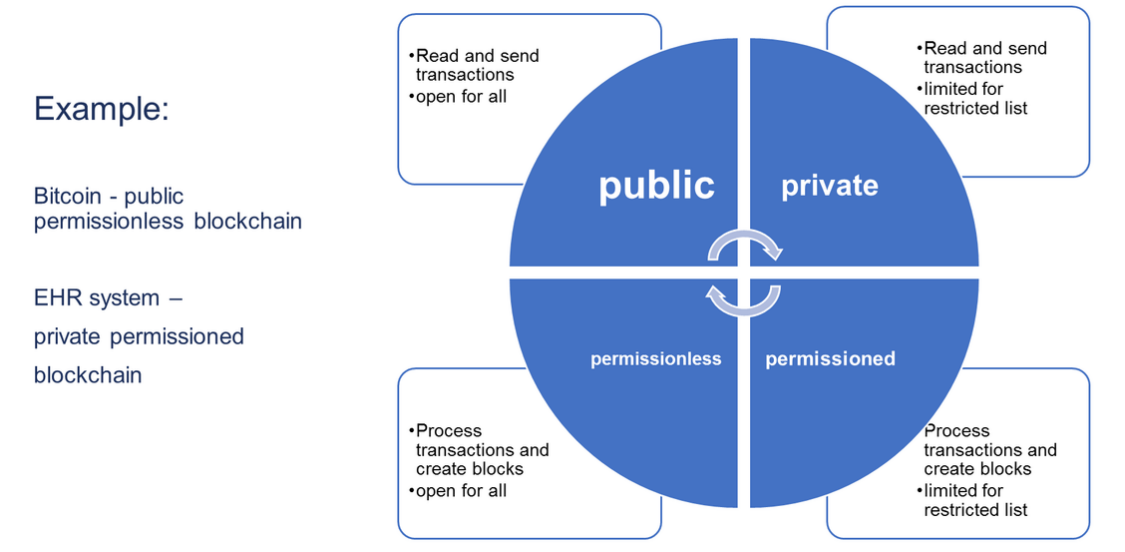
Blockchain types. EHR: electronic health record.

Figure 1 shows the differences between the types of blockchains. In a public blockchain, anyone is free to join and participate in the blockchain network's core activities. A private blockchain allows only the selected entry of verified participants; the operator has the right to override, edit, or delete the necessary entries on the blockchain. A permitted blockchain has the properties of private and public blockchains. Permissioned blockchains have seen an increase in popularity thanks to their ability to allocate specific permissions to various network users. A blockchain database contains information about each transaction of exchange between users. The transactions are verified by the miners, who check the authenticity of the committed actions and then form blocks from these transaction records. Information is distributed on each network member host or the so-called node.

**Figure 2:**
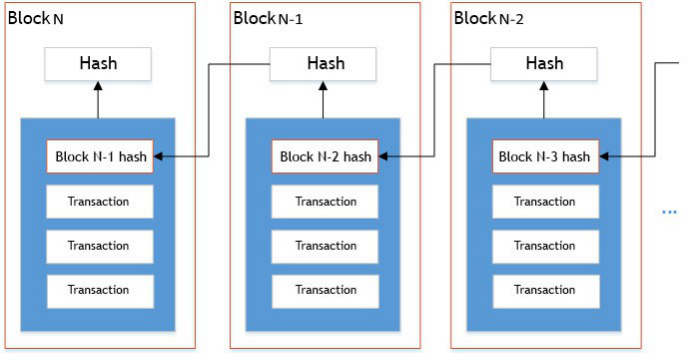
Blockchain hash function.

As Figure 2 shows, a blockchain is a distributed database with a sequence of attached and attaching blocks where every following block includes the value of the previous block's hash function as hash information. All peers of the peer-to-peer network performing information exchange processes have the same sequence of blocks. A long chain called a transaction log is the result of block interconnections [11].

Transactions are connected into blocks, where each block contains nearly multiple transactions that form a hash tree. Further, the block is transmitted to a distributed ledger network where it is checked by specified system participants to avoid mistakes and guarantee correctness.

Providing increased attention to distributed registry technology will lead to an understanding of this technology's potential application in health care systems. Such conditions facilitate the integration of blockchain technology into existing projects, the development of new high-tech ones, and working with a large amount of data [12,13].

Cryptographic tokens are programmable digital units of the value recorded on a distributed ledger protocol such as a blockchain. Tokens do not have their own blockchain but depend or exist on an existing blockchain. Tokens may represent fungible or in-fungible units of value in the form of money, coins, points, digital items, or representations of real-world physical items and rights. Tokens can exist on public/open and permissionless blockchains that anyone on the Internet can view, or they can remain private, such as within an enterprise business network.

The last decade has had a revolutionary impact on the way information is handled. More recently, meaningful information was considerably expensive, and the means for its analysis and dissemination were not available to ordinary citizens. Today, information resource availability has significantly increased, which could be a valuable resource for teamwork and decision-making. Blockchain could significantly benefit telemedicine by managing medical data access and teamwork in organizations. Despite their power, tools based on blockchain are easy to use and do not require high financial costs. The only remaining problems are data interoperability and access restrictions due to imperfect regulatory policies, lack of technical regulation in the field of medical data exchange, and deeply rooted paper-based work traditions in many countries [14-17].

At present, it is possible to represent any information, including numerical data, texts, audios, and images, in a digital format suitable for storage and processing on a personal computer; moreover, the necessary infrastructure for rapid distribution of information is available. For example, we use high-speed mobile Internet technology based on long-term evolution, a wireless broadband communication standard. These technologies make it possible to provide instant access to a vast range of information systems using hand-held personal computers, the functionality of which is contained in most modern mobile phones [18].

There is also a widespread introduction of technologies that will make using digital information ubiquitous. However, in health care, it is significantly inferior compared to other areas, such as banking solutions or retail selling of consumer products. Thus, many promising opportunities for the application of information and communication technologies allowing for increased availability and quality of medical services are currently not implemented to the appropriate extent.

Several reviews on digital technologies to change the health care system landscape have been published lately. These reviews state that blockchain technology offers a platform that could be used for many potential applications in health care. Although this technology is in the early stages of design and development, many organizations have proposed solutions that have the potential to increase health care data transparency and operating efficiency. However, the scalability, security, and cost-effectiveness of blockchain technology will require further research prior to large-scale production and deployment [19-21]. The deployment of blockchain technology in telehealth and telemedicine technology is still in its infancy. Several challenges and research problems must be resolved to enable the widespread adoption of blockchain technology in telehealth and telemedicine systems. The latest survey on telehealth and telemedicine systems shows that they are centralized and fall short of providing the necessary information security and privacy and there is a lack of blockchain-based health care studies considering its use in telemedicine [22]. This topic has attracted considerable interest in the context of the COVID-19 lockdowns. The vulnerable situation created owing to the COVID-19 pandemic has shown the necessity of developing a single-source blockchain-based pandemic health record management system to address several existing and future challenges. Storing, sharing, and accessing COVID-19 pandemic data in a single source of information (database) through blockchain is the most crucial step to address the previously stated challenges; nevertheless, many issues need to be resolved by international health organizations, country leaders/governments, and international policy makers to introduce government-to-government digital health service–related policies, data sharing acts, and health policies. Further, issues regarding digital connectivity, digital inequality, and the digital divide that exists primarily in the least- and under-developed countries around the world must be addressed. This pandemic situation is the perfect opportunity for humanity to bring all countries together regardless of their differences under a single umbrella for ensuring world health safety and fighting against COVID-19 and future pandemics [23-25]. In many cases, remote consultations have become the only option for patients. Our study concerns the practical aspects of implementing available blockchain technology in telemedicine.

## Methods

### Knowledge Review

Our goal was to review the narrow segment of scientific and public domain knowledge regarding the interconnection between telemedicine and blockchain technology. Many successful projects do not have any grounding in research (for example, HapiChain, DocCoin, and others); hence, we decided to include conventional web search engines, where people generally showcase their innovations, often based on personal needs. Most companies in the blockchain space are start-ups based on emerging technologies, whereas the literature is stale owing to the publication lag time (eg, 6-12 months to conduct the research followed by another 3-18 months to publish the findings). In this study, we discovered different types of solutions with similar standard features.

### Search Strategy

The search was based on 2 main source types. The first source was web-based journal databases, indexes, and reference lists. We searched for prototypes and worked in progress using the following search terms: telemedical, telemedicine, blockchain, and distributed ledger. We constructed a search string using AND, the conjunction logical operator, and OR, the disjunction logical operator ([telemedical OR telemedicine] AND [blockchain OR distributed ledger]). The search was based on the metadata, including, title, abstract, and keywords. We targeted original research papers and review articles indexed by PubMed, Scopus, Web of Science, IEEE Xplore, and Google Scholar.

The second source comprised conventional web search engines (Google, Yahoo, and Yandex). The first 100 results from each search engine were analyzed as the most relevant results to find the websites of the relevant projects. We performed a search for whitepapers, system manuals, relevant user information, and other information describing project features on these websites. We searched the web-based journal databases and websites independently of each other. We searched the journal databases first and then searched the related sites.

### Selection Criteria

The main inclusion criterion was that the project must play a role in telemedical service and use blockchain technology as a significant domain. We settled on this criterion to filter out projects based on the opinions of their developers. From a broader perspective, authors can suggest many blockchain-based applications in theory that are suitable for telemedicine services. Our approach was to use only the data about those projects being used in telemedicine. We excluded projects without English-language websites. We also excluded projects without a definite whitepaper or similar information guide. Projects without precise contact data consisting of addresses and phone numbers were also excluded. Publications for the last 10 years only were considered. At least 2 authors checked all the selected projects. Data on similar projects found from different sources were combined.

## Results

### Evaluation and Assessment of Project Features

We analyzed the following features: medical data access (electronic health record [HER] distribution), medical service processing, video conferencing, epidemiology reporting, diagnostic support (with artificial intelligence [AI] technology), treatment support (with AI technology), patient data aggregation (for clinical trials, etc), visit arrangements for medical procedures, ordering medicines from pharmacies, payment processing, and fundraising. These features resulted from the analysis performed by the coauthors on assessing the cross-represented systems. Their significant features were identified, and similar features in different systems were found.

We assessed the suitable projects and recorded their functionalities in a spreadsheet (see Multimedia Appendix 1). We analyzed the function descriptions in the published articles and project website materials.

### Data Analysis Results

We found 37 matches in PubMed, 8 publications in Scopus, 5 in Web of Science, 22 publications in IEEE Xplore, and 547 results in Google Scholar. As for the web search engines, we obtained 118,000 results from Google, 242,000 from Yahoo, and 118,000 results from Yandex. From the first 100 web pages of each search engine and the most relevant results from journals, a total of 36 suitable projects were identified (only 3 of them in journal databases). As the results show, all the identified projects can be divided into the following areas: tracking the origin of data (2/36, 5.6%), storing and managing data (21/36, 58%), telemedicine services (5/36,14%), diagnosing (3/36, 8%), and using blockchain to raise funds (5/36, 14%).

We can describe these areas as follows: The origin of data is an implementation of the authentication procedures based on blockchain. Storing and managing data could be possible using blockchain solutions in different ways. Generally, the most sensitive data could be stored in blocks on the blockchain itself. However, this is almost impossible for significant amounts of data owing to computational difficulties. The best solution is to store data on the cloud, in an encoded manner, with the data key stored in the blockchain. Software solutions could be limited to only the authentication and data transfer provision or could perform as telemedicine service providers themselves with voice- and video-streaming capacities. Another way in which blockchain could help to improve the health care system is through smart contracts. They could be used for automation and control of the diagnostics. If we have a list of procedures to be performed before we are sure of the examination results, we could load this list into a smart contract and perform them.

Moreover, we cannot ignore the financial aspect of this technology. As the popularity of blockchain stems from Bitcoin and other cryptocurrencies, it is used in many cases to sell tokens as a representative of some value. These tokens are proposed to be used to pay for medical services, and they could be earned for clinical trial participation or adherence to the prescribed treatment.

### Selected Projects

Only 18 projects met the selection criteria. The 5 most prevalent features of the available solutions (N=18) were the following: medical data access (14/18, 78%), medical service processing (14/18, 78%), diagnostic support (10/18, 56%), payment transactions (10/18, 56%), and fundraising for telemedical instrument development (5/18, 28%).

The primary role of digital systems based on blockchain technology for telemedicine is to distribute the medical information of patients and provide access to this data for specialists. Usually, the systems working in this direction allow the patient to control which medical data are to be placed in the system and who can access these data. As the implementation of access control does not require any unique technical means or bureaucratic delays, the possibilities that open up to users are extensive [26].

In this study, we examined several examples of currently existing telemedicine systems based on blockchain technology. The Medicalchain project (United Kingdom) mediates patients' consultations with doctors. Medicalchain is based on a double blockchain structure: The first blockchain controls access to medical data and is built using Hyperledger Fabric, whereas all applications and services on the platform run on Ethereum. Any interactions with medical records are recorded as transactions in the blockchain registry [27].

The Symptomatic Platform (United States) was created to support patients with multiple sclerosis, but it is suitable for managing data on any chronic disease. Telemedicine in this system is carried out by video conferencing. Users can store, analyze, and compare their data with others; they can also submit reports, perform statistical evaluation of the epidemiological situation, and undergo genetic screening [28].

The Docademic system (Mexico) facilitates patients’ communication with doctors through videoconferencing. Medical data are stored in the blockchain system. For doctors, the system offers reports on epidemiology, tips on diagnosis, and treatment suggestions. In addition, experts have the opportunity to interact with large groups of patients with similar disease profiles and other characteristics. Furthermore, it can be used by patients to pay for services using cryptocurrency [29].

Robomed (Russia) is controlled and administered by smart contracts based on the Ethereum blockchain. The system aims to provide telemedicine services to patients. The Robomed electronic health record (her) medical information system allows medical organizations to register, connect, and operate on the Robomed network. Robomed's core functions include real-time monitoring of all patient interactions, health care staff decision- making, access privileges, health care professional scheduling, patient health analysis, and consulting services. The Robomed mobile app allows patients to receive telemedicine consultations and exchange EHRs. Using smart contracts, Robomed organizations can track and verify patient health statuses and adhere to clinical guidelines for health care services [30-32].

The DocCoin project (Estonia) has a similar working principle. The user receives access to the service through the “Doc in pocket” mobile app. In the system, doctors receive payment for their services in electronic currency. The system's development began in 2015, with an initial coin offering in 2018 to exchange its tokens for cryptocurrency. Payment is charged for services such as storing medical data, visiting specialists, ordering medicines, and visiting medical institutions [33]. DocCoin is a global service that integrates the entire online medicine industry and offers advantages to businesses and clients. DocCoin provides access to doctors around the world through its smart contracts. Every user can receive specialist advice 24/7 anywhere in the world in any language.

The Memorial Hermann Health Network network helps connect patients and health care organizations, providing them with the ability to independently manage and control their data. This system enables individual organizations to provide data anonymously for scientific research. The system is implemented on the basis of blockchain technology and allows one to create an account for storing personal data; smart contracts provide encryption, and this system is aimed at big data analytics. A blockchain-based solution helps distribute control among stakeholders, which provides strong protection against fraudulent activities. Blockchain also provides transparency, traceability, auditing, and security, and allows data to be identified through decentralized storage of information [34].

The Trusted Health system (United States) has a similar operating principle, currently preparing for the release of its tokens. The accumulated medical information can be used for scientific research. The most significant interest in patient data is expressed by organizations conducting clinical studies, insurance companies, pharmaceutical companies, and organizations providing analytical and consulting services. The presented information about the health of patients helps specialists improve treatment methods, attract clients, increase profits, and reduce expenses while simultaneously reducing the cost of collecting information, thus improving its quality [35].

Another application of blockchain technology is the creation of diagnostic systems. For example, the Skychain project (Russia) is an infrastructure for the placement, training, and use of AI, designed to perform diagnostics. As planned, AI should not replace the doctor but must monitor medical decisions to prevent mistakes. It is supposed to use its cryptocurrency to function, which is necessary to run smart contracts. The proceeds will be received by the owners of the neural networks performing diagnostics. Doctors and patients will be able to evaluate the results of research by several neural networks simultaneously and verify the diagnosis based on the obtained data [36]. This can be done without personal visits to different specialists. Once created, medical images or other diagnostic materials can be evaluated by distant doctors using telemedical services.

The DeepRadiology (United States) project aims to train machine intelligence but in a narrower direction. Medical images obtained by radiological methods are taken into account. This decision is relevant owing to the high degree of subjectivity in evaluating the medical images. The result of the study depends entirely on the qualifications and experience of the specialist evaluating the image. In November 2017, DeepRadiology reported on the first artificial intelligence (AI) system that could interpret computed tomography (CT) with a performance level higher than that of doctors. The system was trained using more than 9 million brain tomography images. The blockchain technology in this system acts to implement a secure storage medium [37]. Blockchain would most effectively integrate as a mode of managing access to sensitive health data. By storing an index of health records and the related metadata linked to the sensitive data (stored elsewhere on a secure cloud), the system would introduce a layer of interoperability to the currently disjointed set of systems.

The eHealth First platform is an international project with the goal of implementing natural language processing as most nonstructured medical records filled out manually. For these purposes, solutions based on AI are also applied. The rest of the system is similar to the previously described ones. It is based on the medical data of patients stored using blockchain technology. It allows associating patients with specialists for diagnosis and consultation. The system also offers researchers solutions, allowing them to conduct research based on accumulated data [38].

In another international system, CareX (United States, Canada, and India), the main emphasis is placed on using the solutions of this platform as a means of international payments. In some cases, the financial transfers through bank payments, even for the payment of medical services from one country to another, raises questions from the financial supervisory authorities in the sending and receiving countries. The remaining functions of the transmission of medical information in this system are also presented. An innovative aspect of this project is the ability to communicate with the chatbot, which implements AI elements for making a preliminary diagnosis [39].

Similar issues affect the operation of the Solve.Care system (Estonia and Ukraine). It allows medical organizations, insurance companies, and patients to work without intermediaries to guarantee payment and provision of services. They are using their tokens for payment [40]. In this group of systems, blockchain technology serves as a supporting service for telemedical interactions.

HealPoint (United States) is based on the Ethereum platform for the implementation of telemedicine services. The system helps patients use medical consultation services, transfer symptoms, medical records, and vital signs of the patient. Ethereum-based smart contracts allow patients to obtain a second opinion from several medical experts around the world. Before providing medical services, network specialists verify the doctor's identity and license. All interactions with patients are digitally signed before being recorded on the blockchain for audit purposes [41].

HapiChain (France) exploits blockchain technology to improve the security, scalability, and reliability of medical workflows. Although HapiChain is patient-centric, it also helps clinicians save time and prevent unnecessary trips without improvising treatment. In HapiChain, two primary telemedicine services are embedded, namely telemonitoring and teleconsultation. HapiCare, an existing health care monitoring system with self-adaptive coaching using probabilistic reasoning, is used for telemonitoring. HapiChain then completes this service by adding teleconsultation services exploiting blockchain technology [12].

High interest in solutions based on cryptocurrency implemented in blockchain technology causes widespread projects to attract investment. This approach allows researchers to fully maintain control without transferring the right to make decisions to a critical investor, board of directors, or any other collegial body formed by financing participants. For example, Elcoin (Russia) aims to obtain funding to develop medical equipment through the release of its tokens. The company has created several medical devices. Funds are needed for further development, which the company collects by electronic emission of tokens. The primary target audience is foreign investors [42]. Therefore, blockchain technology can be used as a medium for fundraising to develop telemedical services.

Technical solutions are also used for the noninvasive diagnosis of diabetes, stomach ulcers, and lung cancer. The Health Monitor project (Czech Republic) is based on the principle of analyzing markers of biochemical processes in the body; in a solution created by system developers, the marker is a mixture of gases exhaled by humans [43]. Furthermore, this research project primarily uses cryptocurrency-based infrastructure as a tool to search for investments.

More unusual is the PointNurse system (United States), which is primarily aimed at telemedicine services, establishing a visual link between specialists and patients. However, it focuses mainly on nursing staff, which is its singular goal. It allows practicing nurses and members of the support team to conduct direct consultations on primary health care to make a health assessment. The system also has its cryptocurrency used to pay for services and encourage patients to follow the recommendations. The system works with specialists in several languages. There is a rating for specialists, and patients can choose whom to contact [33,44,45].

MedCredits (United States) uses Ethereum to help doctors diagnose dermatological patients using telemedicine. It is a secure system that protects users from intruders through the implementation of reputation-based systems. Moreover, it allows one to check doctors' licenses. Two Ethereum-based smart contracts implemented in MedCredits help automate escrow-protocol-based payments and validate medical services. The doctor can access the patient's symptoms to diagnose and prescribe treatment using the blockchain [33,46].

The common feature of blockchain-based services is that they deal with sensitive private data that can affect patients’ health. Therefore, security and privacy issues should be solved first. Blockchain technology–based systems can quickly provide suitable solutions, as observed in clinical trials [47].

## Discussion

### Telemedicine Services

Telemedicine services, including radiology, dermatology, and cardiology services, could be provided in different ways to help chronic patients and monitor acute patients. Real-time telemedicine (also called live telemedicine) makes it easy to facilitate doctor-patient interactions anytime and anywhere. Live telemedicine includes videoconferencing and telephonic consultations that let providers and patients communicate in real time. Assessments of medical histories, essential visual examinations, psychiatric evaluations, and even ophthalmic tests can all be conducted via real-time telemedicine. Remote patient monitoring allows health care providers to monitor patients’ health data remotely, usually when they are in their own homes. Remote patient monitoring is especially effective for chronic conditions, ranging from heart disease to diabetes to asthma. Telemedicine can improve communication among the members of a medical team. A primary physician can get greater access to a wide range of specialists without requiring any travel. Telemedicine technology has considerably accelerated the rate at which X-rays, computed tomography scans, and other important images are distributed from one medical professional to another. Thanks to telemedicine, health care professionals have multiple ways to interact with patients in their own homes. Web-based services, such as patient portals, allow providers to share essential information and answer simple questions. Telemedicine could be the only option in harsh conditions; for example, space stations and polar expeditions could receive only remote professional medical help. The outbreak of COVID-19 proved that telemedicine was an effective option to fight a pandemic [24].

For example, a patient can obtain advice from a specialist without visiting another clinic or making a trip to a remote city or country. There is no need to remain on the waiting list; systems with a large pool of doctors instantly allow patients to find the required specialist. The expansion of such systems to the financial sector can also be implemented through blockchain transfers using cryptocurrencies. At the same time, within the framework of the smart contracts, participants are guaranteed the provision of services on the one hand and payment on the other. Smart contracts are pieces of code that sit on the blockchain. Once a smart contract is executed, payments are automatically deducted from a patient's digital wallet, and funds are moved into the supplier's digital wallet; therefore, payment happens seamlessly. Inside a smart contract, exact rules could be included, describing the conditions such as when payments will be transferred. It could be an acknowledgment of some medical data or procedure completion.

### Smart Contracts and Tokens

Blockchain technology can facilitate such an infrastructure in the form of a decentralized marketplace where access to health data is under the individual's control. Information seekers can post their queries and individuals can remain anonymous and decide whether they want to share their data. With the tokens in a blockchain-based marketplace, a reward can be automatically transferred based on a digital contract once the data has been delivered. Such a system has a clear advantage over a fiat currency–based system where an agent must always be involved, and the large population of unbanked individuals cannot participate.

Diagnostic centers can be included in the same unified system; following the recommendation of a physician made via a telemedical service system, an appointment can be scheduled for a specialized examination using inpatient diagnostic equipment that requires an on-site visit. The same principle applies to working with pharmacies. Pharmacy institutions connected to the system will receive a guarantee that this drug was prescribed by the physician who referred to the patient indicated in the system. The high degree of transaction security in blockchain systems ensures a suitable level of data authenticity.

Smart contracts are extremely useful for telemedicine. They are specific computer codes built into the blockchain network and are executed on computers or nodes. Terms between the parties in the smart contract are written in the form of code in the blockchain. The involved parties are anonymous, but the contract itself has available public properties. When a starting event has happened, for example, the occurrence of a specific date, the contract launches itself based on the conditions of the provision recorded in its code. Following the terms of the contract, the network nodes update the register. After all the requirements are met, the contract is automatically closed, and information about the actions performed is recorded in the blockchain.

In this work, 18 blockchain systems for telemedicine were analyzed. The results are presented in Multimedia Appendix 1.

Their capacities were summarized and systematized under a common methodology to identify suitable opportunities for applying blockchain technology to provide possible telemedicine services. To determine the required properties, the role of telemedicine services in the health care system has been evaluated. Furthermore, the role of digital systems based on blockchain technology for telemedicine has been determined. The most promising projects available on digital support of telemedicine services using blockchain technology are presented in this paper.

Analysis of existing solutions has shown advantages for patients, medical organizations, and related institutions. Telemedicine solutions can increase the availability of medical services for patients, reduce the burden on medical institutions, reduce the cost of providing services, and increase their delivery efficiency. Telemedicine does not require expansive facilities and can optimize the utilization of on-site medical staff resources. Blockchain technology addresses the issue of access to medical data and the preservation of their confidentiality. Another reason for blockchain system implementation is its financial capacity. It will ease the transfer of payments for medical services from one country to another without bureaucratic delays. It could be used as a payment method from one medical institution to another, from a hospital to a foreign bank, or from a patient to a foreign hospital. Nevertheless, all participants need to have blockchain system tools to achieve this objective or employ brokers to convert blockchain tokens to fiat money. In addition, governmental control and audit of foreign exchange inflows and outflows will be needed.

### Blockchain and Telemedicine Drawbacks

Despite general concerns against blockchain as with any new technology, these solutions are successful at the pilot level. If we try to address only the most common issues, namely decentralization risks, expenses, and computational complexity, blockchain can become a unified (but decentralized) medical information database. In future, it will be possible for doctors to fill this database not only from EHRs but also with Internet of Things gadgets. The blockchain can also record the results of group examinations from diagnostic centers and information about clinical trials of drugs. Any hospital in the country will have authorized access to patient data. This will enable doctors to share clinical trial results faster, which will accelerate the development of drugs for severe diseases. Centralization in this perspective will create eminent dependency on a central node. This will result in a bottleneck for the entire system.

In telemedicine, the main issue is medical data protection. Additional effort is required to prevent unauthorized access to patients’ medical data, especially if they access telemedicine on a public network or via an unencrypted channel. One of the main challenges faced by blockchain solution developers is securing the blockchain. For example, in Bitcoin, miners use their computing systems that consume electricity to perform the computations required to verify data on the blockchain. In return, they receive a reward in the form of digital money.

Projects not related to cryptocurrencies cannot provide network participants with a similar form of reward. Therefore, it is difficult for them to generate public interest. In telemedicine systems, the information in the blockchain can be protected by medical organizations and research agencies.

They will act as miners and use their hardware and computing infrastructure to maintain the integrity of the blockchain. In return, the system will provide them with access to anonymous patient data (with the latter's consent) for epidemiological and other research activities.

For example, such a project has already been implemented in Estonia [48]. Since 2008, all hospitals and doctors in the country have been mandatorily digitizing health information. Recently, blockchain has become responsible for its own security.

The distributed ledger acts as a database for medical records. When changes are made to a patient's medical record, this event is immediately recorded on the network, along with information about what exactly was changed, deleted, or added. This transparency level allows therapists, surgeons, pharmacists, and other professionals to receive up-to-date and correct patient information acceptable to the community.

The system makes it possible to make more accurate diagnoses, taking into account a fully documented medical history, guide doctors in crises (for example, provide information about allergic reactions to drugs), and even adjust treatment for chronic diseases over time (depending on changes in the patient's condition).

### Conclusions

Our study identified several blockchain technology projects to provide telemedical services. They differ considerably from each other and should be examined separately. There is no “silver bullet” in this market, but we suggest some points of interest to start with in our review. These various features (medical data access, medical service processing, epidemiology reporting, diagnostic support, and treatment support) allow us to talk about the possibilities of integrating blockchain technology into telemedicine and health care on different levels. In this area, a wide range of tasks can be identified that could be accomplished based on digital technologies using blockchains. Almost all existing projects are prototypes that are under development. Considering that many of them are aimed at attracting investments, it can be said that the role of scientific, technical, legal, and economic experts in assessing the feasibility of investing in various start-ups based on blockchain technologies is increasing.

Solutions based on blockchain technology are of most interest for countries where there are currently no working centralized information processing systems used for telemedicine. Launching a distributed system based on open-source solutions provides a potential opportunity to avoid significant investments in building a centralized system.

## Appendix

Multimedia Appendix 1 Functionalities of the suitable blockchain-based projects analyzed in this study.

## Abbreviations

AI: artificial intelligence
EHR: electronic health record
